# Role of *trans*-resveratrol in ameliorating biochemical and molecular alterations in obese rats induced by a high fructose/fat diet

**DOI:** 10.1038/s41598-025-91027-7

**Published:** 2025-03-06

**Authors:** Marwa Maher Khamis, Said Salama Moselhy, Shaimaa Rihan

**Affiliations:** Biochemistry Department, Faculty of Science, Ain Shams University, Cairo, Egypt

**Keywords:** *trans*-resveratrol, SIRT-1, PGC-1α, Cyto-c, GLUT-4, Rats, Biochemistry, Molecular biology

## Abstract

We evaluated the effect of *trans-*resveratrol (RSV) in ameliorating biochemical and molecular alterations in obese Wister male rats fed on high-fat/high-fructose-fed. Male Wister rats were divided into eight groups and fed with either a standard diet (control), high fructose (HF), high fat (HFAT), or a high- fructose high- fat (HF/HFAT) diet and supplemented with RSV (30 mg/kg/day) for 4 weeks. The food intake, body weight, glycemic parameters, lipid profile, oxidative stress were assessed. SIRT1 gene expression, PGC-1α, cyto-c and GLUT-4 were evaluated by qRT-PCR in adipose tissue of normal and obese rats. The body weight gain, serum fasting glucose, insulin, and HOMA-IR values were significantly higher in the HF and HF/HFAT groups than in the HFAT and control groups. Hyperlipidemia was observed in high calorie diets fed rats compared to control group. The levels of total cholesterol, triglycerides and LDL-c were significantly elevated while HDL- c was significantly decreased in HF & HF/HFAT groups compared to HFAT group. The levels of serum malondialdhyde (MDA) and superoxide dismutase (SOD) activity in adipose tissue were elevated in all groups compared to control group, particularly in the groups that were kept on a high fructose diets (HF, HF/HFAT). SIRT-1, PGC-1α, Cyto-c, and GLUT-4 genes levels were significantly down regulated in HF, HFAT & HF/HFAT groups compared to control group. Supplementation of T-RSV restored the alteration in carbohydrates-lipid metabolism as well as oxidative stress and upregulation of SIRT-1, PGC-1α, Cyto-c, and GLUT-4 genes. RSV is a promising treatment in the management of pathologic consequences of obesity from high-calorie diet consumption via molecular alteration of target genes.

**Supplementary Information:**

The online version contains supplementary material available at 10.1038/s41598-025-91027-7.

## Introduction

The obesity is now recognized as one of the most crucial public health issue. It was reported that the global adult obesity rate has more than doubled since 1990, and adolescent obesity has quadrupled. In 2022, 43% of adults worldwide were overweight (body mass index [BMI] ≥ 25 kg/m^2^) and 16% were living with obesity (BMI ≥ 30 kg/m^2^)^1^. The obesity is related to decreased life expectancy due to increased mortality from chronic diseases, including type 2 diabetes mellitus (T2DM), hypertension, cardiovascular disease, and some cancers^2^. In addition, obese individuals have high risk for severity and mortality in COVID-19 and influenza infections^3^.

Different factors contribute to the development of obesity including genetic, behavioral, social, and environmental that lead to abnormal fat accumulation, from insufficient energy expenditure^4^. Processed foods contain high fat and refined sugar, especially fructose, which are related to increased prevalence of obesity^5^. Obesity represents symptoms described as an exaggerated accumulation of fats in adipose tissue caused dyslipidemia, development of coronary heart disease and consequently reducing the life expectancy^6,7^.

Experimental animal studies are used for investigating changes in morphology and metabolic pathways that contribute to the onset of overweight and obesity. Typically, a high-fat semi-synthetic diet (HFD) with 45–60% of total calories is used to create animal models for diet-induced obesity (DIO). It was reported that, the use of diet with 45% of kcal present as fat in combination with a high mono or di-saccharide content (fructose or sucrose) has been advocated for DIO models^8,9^. The experimental period may affect different organ functionality caused by the dietary components for development of obesity^10,11^.

Medications for the management of obesity are lmited due to their potential harmful side effects. The natural product supplements are the first line in keeping good health by preventing diseases and their complications^12,13^. The anti-obesity effects of herbal formulations are given under clinical supervision to avoid any complications. The usage of medicinal plants and their derivatives is an approach for managing obesity and related diseases^15^. Resveratrol (3,5,4′-trihydroxystilbene) is a polyphenolic compound that is present in many different types of plants. It is naturally found as *trans*-or *cis*-stereoisomeric forms, but *trans*-isoform is more biologically active^16^. Naturally, resveratrol can be found in a variety of foods such as peanut, pistachios, blueberries, green and black grapes, raisins, and grape juice^17,18^. Clinical trials were conducted using varying dosages, durations, and methods of administration of resveratrol, ranging from 5 mg to 5 g, depending on the patient’s health status^19^. In addition, several studies have extensively evaluated the role of resveratrol in combating oxidative stress, obesity, diabetes, cancer, microbial infections and inflammatory, heart and neurological diseases^20–24^.

The hypothesis of this study to identify the modulation of SIRT-1, PGC-1α, Cyto-c, and GLUT-4 ) expression by RSV as a mechanism of abrogation the alterations caused by high caloric intake.

In this study, we developed an animal model of DIO, by feeding a HFD (45% of total dietary Kcal) and refined sugars (25% fructose solution), to mimic obesity in human population. The aim of the present study was to investigate the effect and mechanism of action of T-RSV in modulating biochemical and genetic alterations (SIRT-1, PGC-1α, Cyto-c, and GLUT-4 ) in obese rats induced by HF, HFAT & HF/HFAT diets.

## Materials and methods

The handling of Wister rats were done following approval from the Ethical Committee at Faculty of Science, Ain Shams University, Egypt. RSV (purity ≥ 98%) was purchased from Xuanchrng Quality Herb Co., China. It was dissolved in an aqueous solution of carboxymethyl cellulose (ASHLAND SPECIALTIES, France) at fed at a dose of 30 mg/kg b.w.

### Experimental design

The study was conducted according to the guidelines as described by the ARRIVE guidelines. Ninety six male Wister albino rats (113.9–136.3 gm) were obtained from VACSERA (Cairo, Egypt). They were kept in standard housing with a 12-hour light/dark cycle, a temperature of 22 ± 6 °C, a humidity of 40 ± 10%, and unlimited access to food and water. After one-week of acclimation, rats were randomly divided into eight groups with 12 animals each: group 1 (Control): Rats were received normal diet, group 2 (HF): Rats were received standard diet and 25% fructose in drinking water, group 3 (HFAT): Rats were received high fat diet, group 4 (HF/HFAT): Rats were received high fat diet & 25% fructose in drinking water, group 5 (Control-RSV): Rats were received normal diet and given orally 30 mg/Kg BW of RSV, group 6 (HF-RSV): Rats were received normal diet and 25% fructose in drinking water with oral doses of (30 mg/Kg bw) of RSV, group 7 (HFAT-RSV): Rats were received high fat diet with oral doses of 30 mg/Kg bw of RSV & group 8 (HF/HFAT-RSV): Rats received high fat diet and 25% fructose in drinking water with oral doses of 30 mg/kg bw RSV. The RSV dose was given according to Macarulla et al.^25^. All rats received the diets daily according to their group for 24 weeks then subsequent RSV supplementation for 4 weeks. The composition of normal diet and high-fat diet that were used in this experiment is shown in Table 1. The HFD was prepared according to diet formula D12451 by mixing (per 100 g diet ; 24 g protein, 41 g carbohydrate, 24 g fat).

**Table 1:** The composition of diets used in the experimental work.

The normal diet	The high fat diet
Standard pellet diet which consisted of carbohydrate 48.8%, protein 21%, and fat 3%, calcium 0.8%, phosphorus 0.4%, fiber 5%, moisture 13%, and ash 8%	Diet pellet consisted of carbohydrate 35% kcal, protein 20% kcal, and fat 45% kcal trans-fat, calcium 0.8%, phosphorus 0.4%, fiber 5%, moisture 13%, and ash 8%

At the end of the experiment, the animals were fasted overnight. Blood and tissues were collected from all groups including RSV treatment after scarification by decapitation under anesthesia with thiopental (15 mg/kg). Blood was collected in vacutainer tube and centrifuged at 2500 rpm for 10 min at 4 °C. Serum samples were then separated for the biochemical analysis. The epididymal white adipose tissue was removed and stored at − 80 °C for analysis.

### Body weight and organ weights

The rats were checked daily for any changes in skin ulcer, eye color and behavior. After collection of blood samples, the animals were euthanized by exsanguination and subjected to complete necropsy. Then, the weights of the organs were measured.

### Biochemical analysis

The fasting blood glucose was measured by an automated enzymatic method using commercial kit (Cat. No. GL 1320, Biodiagnostic, Egypt). Commercially available ELISA kit was used to assess serum levels of insulin (Cat. No. MBS724709, MyBioSource, USA). Homeostatic model assessment of insulin resistance was calculated from the following equation: HOMA-IR= [fasting insulin (µU/mL) × fasting glucose (mmole/L)/ 22.5]^26^.

Triglycerides (TGs), total cholesterol (TC), LDL-c and high-density lipoprotein cholesterol (HDL-c) concentrations were determined using enzyme-based assay kits (Cat. No. TR2030, CH1202, CH1231, and CH1230, Bio- diagnostic, Egypt). The level of Malondialdehyde (MDA) and the activity of superoxide dismutase (SOD)( MBS2540402).

were measured by Colorimetric Assay Kit from MYBioSource (MBS2540407).

### Gene expression analysis

Quantitative RT-PCR was used for target genes expression assay by SYBR green (CAT. NO. 204141, Qiagen, Germany) (Agilent Stratagene MX3000P, USA). The primer sequences of following genes SIRT-1, PGC-1α, Cyto-c and GLUT-4 are shown in Table 2(Macrogen, Korea).

**Table 2:** PCR primers used in all quantitative PCR assay.

Gene	Forward sequence (5´→3´)	Reverse sequence (5´→3´)	Annealing temp. (°C)	Product size (bp)
SIRT-1	TCGGCTACCGAGACAACCTC	AGCTTGCGTGTGATGCTCT	61	99
PGC-1α	GGTGAGGACCAGCCTCTTTG	TATGTTCGCGGGCTCATTGT	60	159
Cyto-c	GAAGAAGGGAGAAAGGGCAGAC	AATCGGGGCTGTCCAACAAAA	61	181
GLUT-4	TCCCCCTCAAGCCCATCTCA	CCACCCACAGAGAAGATGGC	60	368
ß-Actin	CACGACACCAAGGGATCTGG	CCCGGTTGATTTTGGGGTTG	60	59.7

The Epididymal white adipose tissue (eWAT) was homogenized and lysed using RNeasy Mini Kit (CAT. NO.74104, Qiagen, Germany) to isolate total RNA following the manufacturer’s instructions. The isolated RNA (500 ng) was reversely transcribed to cDNA according to the method of (Romestaing et al., 1997) with a cDNA reverse transcription kit (CAT. NO. 205311, Qiagen, Germany). The expression of mRNA was determined by the ^2−ΔΔ^CT method^27^.

### Statistical analysis

All the data were presented as mean ± SD and analyzed using one-way analysis of variance (ANOVA) and Tukey’s post hoc test with IBM SPSS statistics version 29.0.2.0. A *p* value < 0.05 was considered as statistically significant difference.

## Results

### Effect of RSV and high calorie diet on body weight changes

**Fig. 1:**
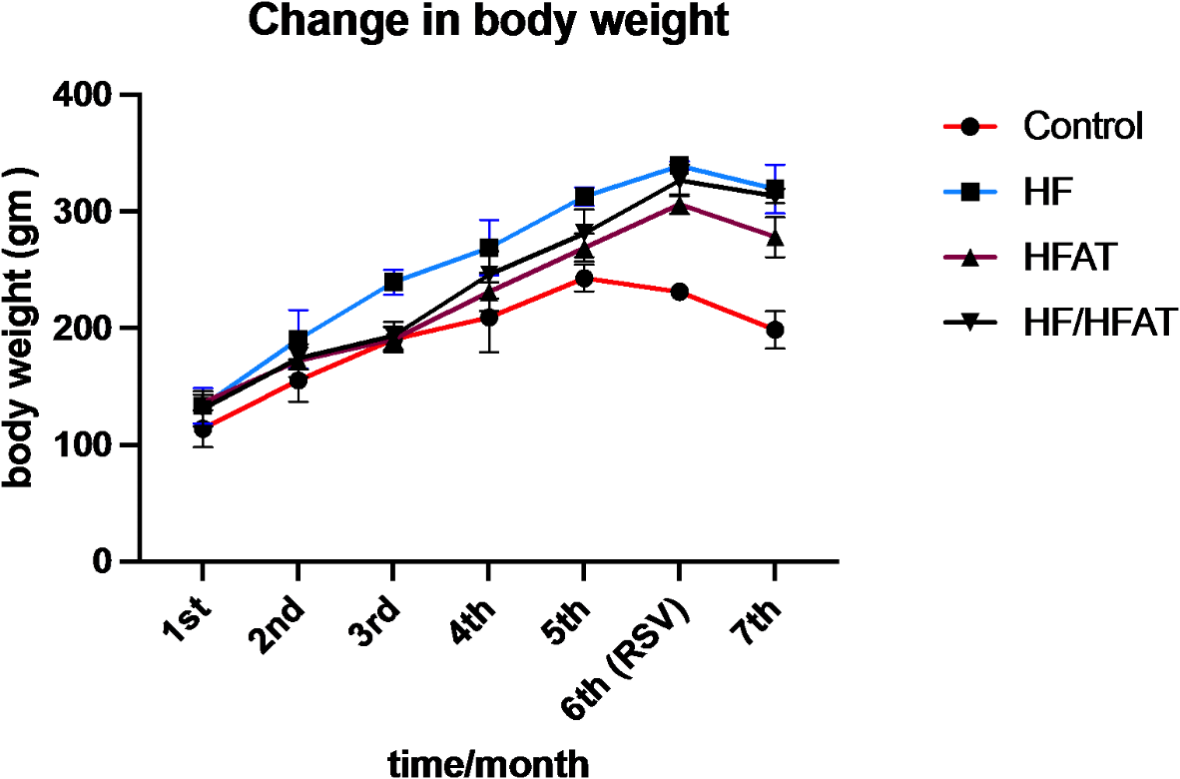
Changes in body weight (g) in all experiment groups before & after RSV ingestion. Data are expressed as mean ± SD. –ve Control: normal diet fed rats; HF: high fructose fed rats; HFAT: high fat fed rats; and HF/HFAT: high fat and fructose fed rats.

**Fig. 2:**
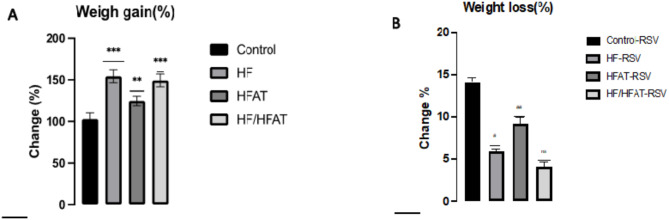
Change in body weight (%) in all experimental groups before and after RSV ingestion. (**A**) Percentage of weight gain before RSV ingestion. (**B**) Percentage of weight after RSV ingestion. Data are expressed as mean ± SD and analyzed by multiple T-Test. *p < 0.05,**p < 0.01, ***p < 0.001 indicate significant difference compared to the (initial weight); #*p* < 0.05, ##*p* < 0.01, ###*p* < 0.001 indicate significant difference compared to the (+ ve control); NS, no significant difference. –ve control: normal diet fed rats; HF: high fructose fed rats; HFAT: high fat fed rats; HF/HFAT: high fat and fructose fed rats; +ve control, normal diet fed rats treated with RSV; HF-RSV: high fructose fed rats treated with RSV; HFAT-RSV: high fat fed rats treated with RSV; and HFHF-RSV: high fat and fructose fed rats treated with RSV.

The changes in body weight were recorded throughout the experimental period to assess the effect of RSV in HF, HFAT, HF/HFAT animal groups. After 6 months, HF-fed and HF/HFAT-fed showed a significant increase in body weight (percent changes 154.34%, *p* < 0.001; 149.35%, *p* < 0.001) in comparison to HFAT-fed group which showed (124.63%, *p* < 0.01). On the other hand, RSV treated rats showed a significant decrease in body weight compared with untreated. In addition, HFAT-RSV group showed a significance reduction in body weight (-9.19%, *p* < 0.01) relative to HF-RSV group (-5.93%, *p* < 0.05) whereas HF/HFAT-RSV group showed no significant changes in body weight reduction (-4.11%,) (Figs. 1 and 2).

#### Morphological alterations

**Fig. 3:**
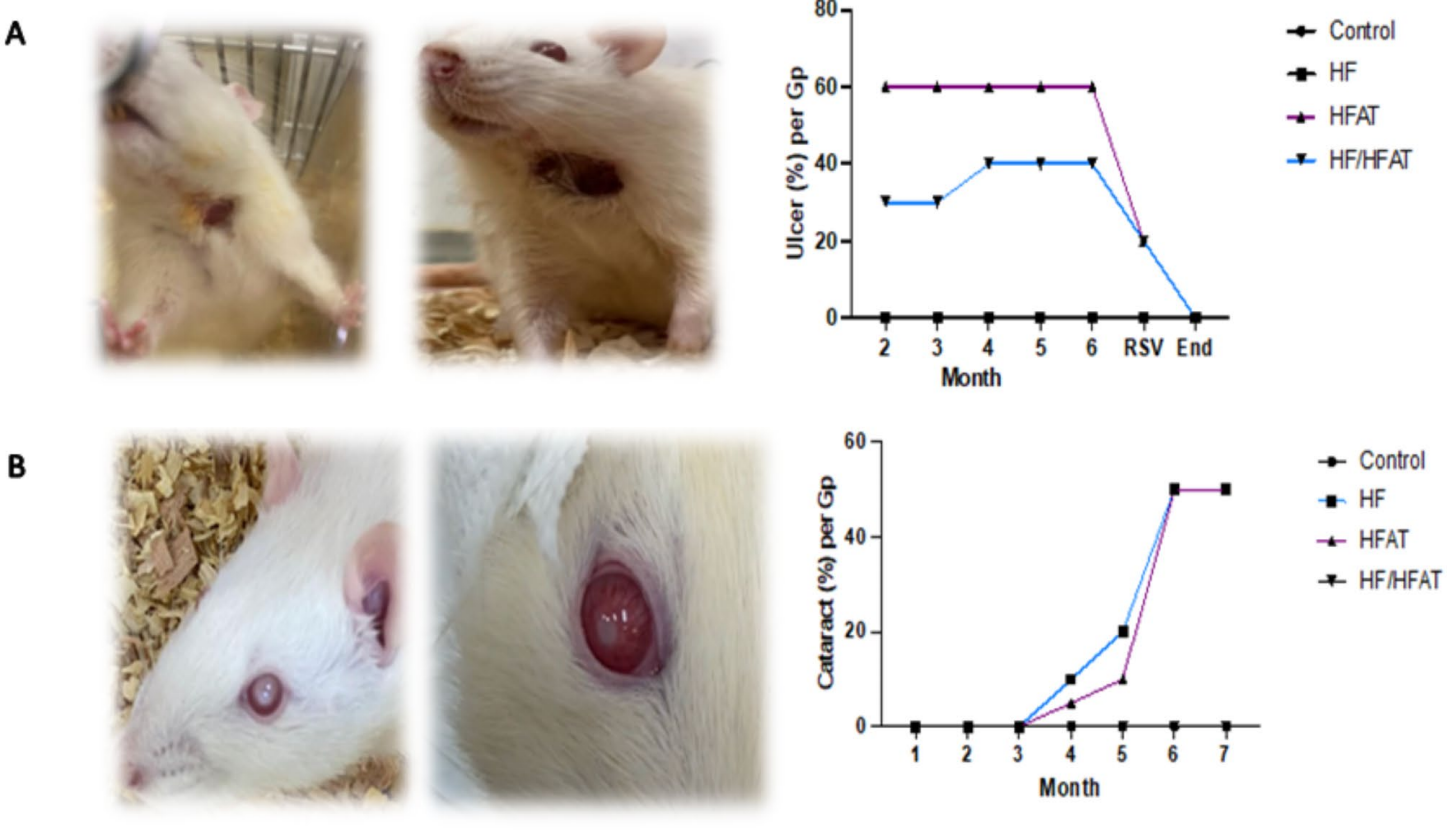
The pathological findings obtained in HF & HFAT groups. (**A**) Animals of developed skin ulcers. (**B**) Animals of developed cataract.

Rats fed on HFAT or HF diet exhibited pathological changes such as cataract (whiteness of eye ball) and skin ulcer. The ulceration of skin started aggressively in HFAT group after two months of high fat diet by 60%, then started to decrease by intervention using RSV to reach 20%. In HF/HFAT group, ulcer started with intensity (30%) then reached 40% at the last three months and decreased to 20% after RSV supplementation. However, 10% of rats fed on HF developed early cataracts and reached to 50%. Alternatively, it began in 5% of HFAT fed rats and increased to 50% at the end of experimental period (Fig. 3). Oral RSV supplementation ameliorates the appearance of eyeball and skin lesions.

#### RSV on internal organs weight

**Fig. 4:**
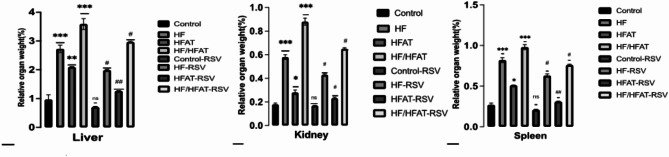
Effect of RSV on relative weights of; (**A**) Liver; (**B**) Kidney; and (**C**) Spleen Data are expressed as mean ± SD and analyzed by multiple T-Test. *p < 0.05,**p < 0.01, ***p < 0.001 indicate significant difference compared to the –ve control; #*p* < 0.05, ##*p* < 0.01, ###*p* < 0.001 indicate significant difference compared to the (+ ve control); NS, no significant difference. –ve control: normal diet fed rats; HF: high fructose fed rats; H-FAT: high fat fed rats; HF/HFAT: high fat and fructose fed rats; +ve control, normal diet fed rats treated with RSV; HF-RSV: high fructose fed rats treated with RSV; HFAT-RSV: high fat fed rats treated with RSV; and HFHF-RSV: high fat and fructose fed rats treated with RSV.

The high calorie diets affected relative weights of the liver, kidney, and spleen. In both HF/HFAT & HF groups, there was a significant increase in the relative weights of the liver, kidney, and spleen (*p* < 0.001)compared with control. However, the HFAT group showed less significant changes in relative weight of these organs. Oral supplementation of RSV improved the relative weight in HFAT group (*p* < 0.01) rather than HF and HF/HFAT groups (*p* < 0.05) versus untreated rats (Fig. 4).

### Biochemical analysis

#### Impact of RSV treatment on the blood glucose level and HOMA-IR

**Fig. 5:**
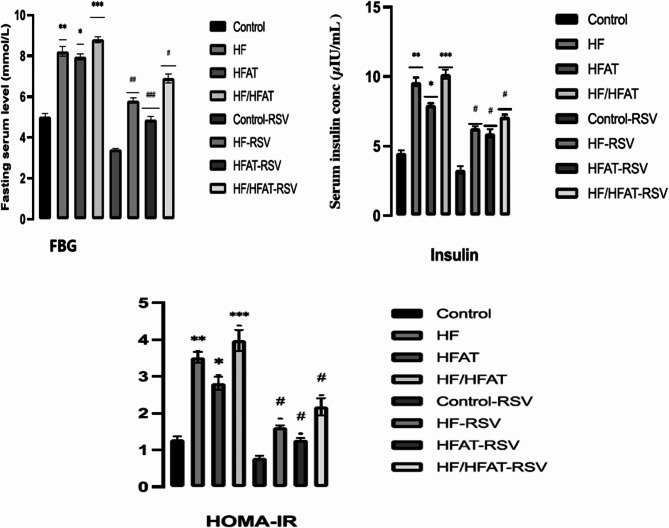
The changes in FBG (mmol/L), fasting insulin levels in serum (µIU/mL); and HOMA-IR for the different experimental groups. Data are expressed as mean ± SD and analyzed by multiple T-Test. *p < 0.05,**p < 0.01, ***p < 0.001 indicate significant difference compared to the –ve control; #*p* < 0.05, ##*p* < 0.01, ###*p* < 0.001 indicate significant difference compared to the (+ ve control); NS, no significant difference. –ve control: normal diet fed rats; HF: high fructose fed rats; H-FAT: high fat fed rats; HF/HFAT: high fat and fructose fed rats; +ve control, normal diet fed rats treated with RSV; HF-RSV: high fructose fed rats treated with RSV; HFAT-RSV: high fat fed rats treated with RSV; and HFHF-RSV: high fat and fructose fed rats treated with RSV.

The fasting blood glucose level in the HF/HFAT and HF groups was significantly higher (*p* < 0.001; *p* < 0.01), respectively than in the HFAT and control groups (Fig. 5). Taking into consideration, weight gain developed at a higher rate in the animals received the HF and HF/FAT diet compared to the HFAT group (339.13 ± 13.57 g; 326.41 ± 11.26 g respectively). Insulin resistance measured as HOMA-IR values were significantly higher in the HF and HF/HFAT groups than in the HFAT group. However, the administration of T-RSV (30 mg/kg) significantly improved HOMA-IR and glucose homeostasis in all groups (*P* < 0.05).

#### RSV treatment on levels of lipid profile

**Fig. 6:**
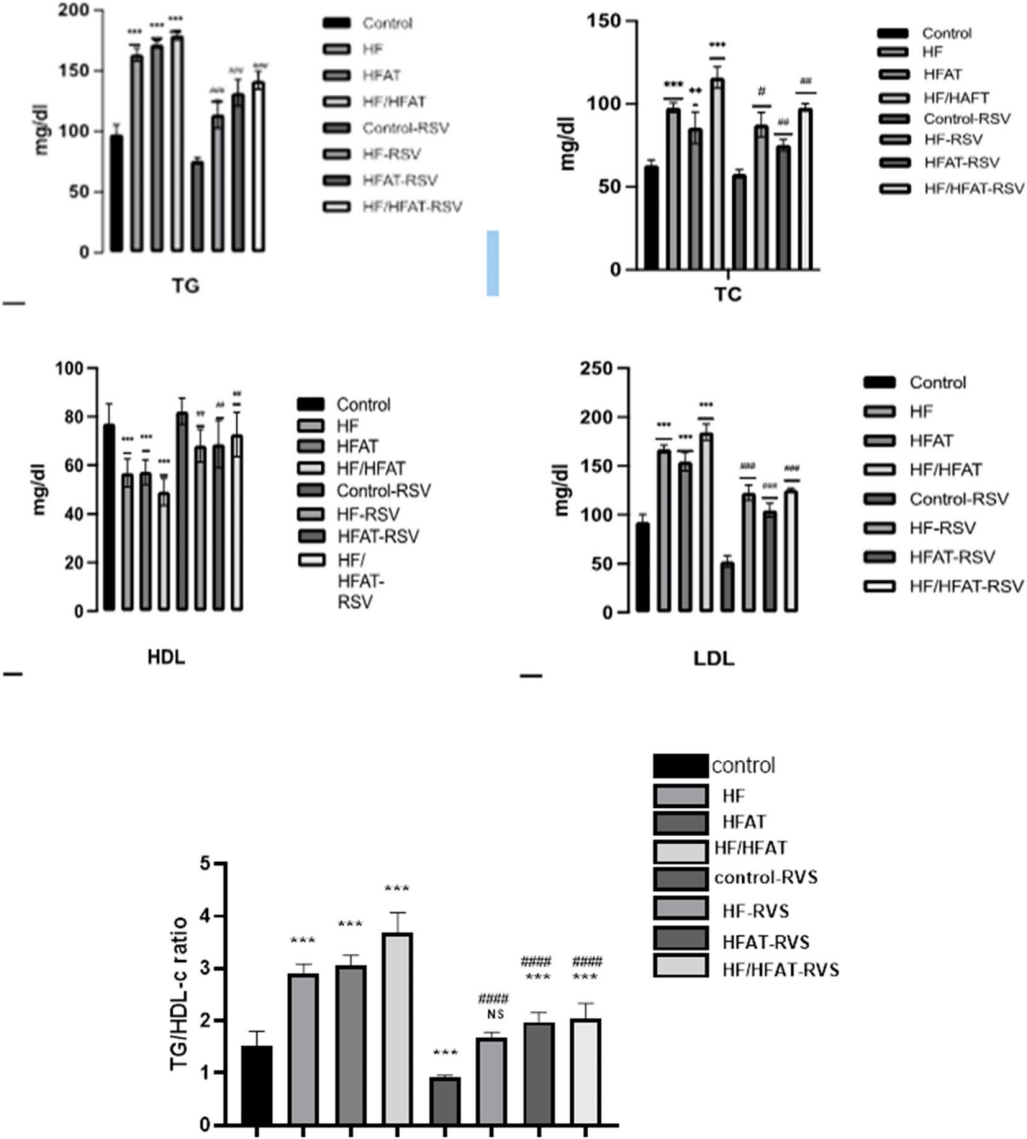
Comparison of RSV effect on Lipid profile for the different experimental groups. The data express the fasting serum level of TC; TG, LDL-C; and HDL-C. Data are expressed as mean ± SD and analyzed by multiple T-Test. *p < 0.05,**p < 0.01, ***p < 0.001 indicate significant difference compared to the –ve control; #*p* < 0.05, ##*p* < 0.01, ###*p* < 0.001 indicate significant difference compared to the (+ ve control); NS, no significant difference. –ve control: normal diet fed rats; HF: high fructose fed rats; H-FAT: high fat fed rats; HF/HFAT: high fat and fructose fed rats; +ve control, normal diet fed rats treated with RSV; HF-RSV: high fructose fed rats treated with RSV; HFAT-RSV: high fat fed rats treated with RSV; and HFHF-RSV: high fat and fructose fed rats treated with RSV.

The high calorie diets fed rats especially HF/HFAT group revealed a significant elevation in lipid profile and hyperlipidemia, reflected by a remarkable elevation in the serum TG, TC, VLDL-c, and LDL-c levels accompanied by a significant decline in high- serum HDL-c compared to normal diet consuming the rats (control). However, the serum TG, TC, and LDL-C levels in the T-RSV groups exhibited a marked reduction with a notable elevation in HDL-C levels, suggesting that RSV intervention could effectively mitigate the adverse effects of a HFD on blood lipid levels (Fig. 6).

#### RSV supplementation on oxidative stress biomarkers

**Fig. 7:**
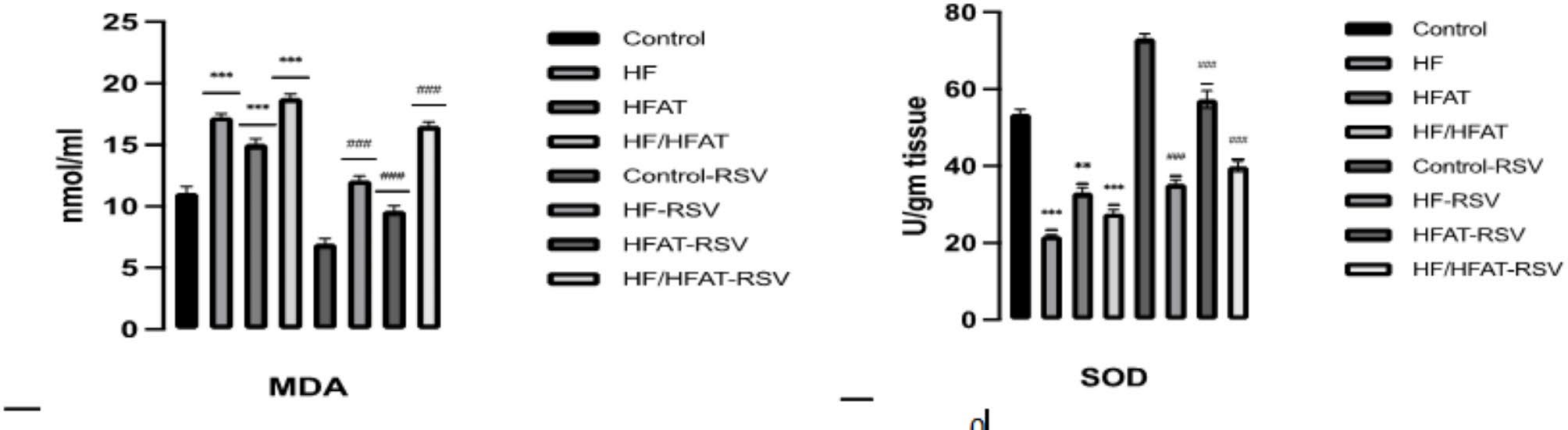
Effect of RSVon the serum lipid peroxidation (MDA content) & SOD activity of epididymal adipose tissue in all experimental rats. Data are expressed as mean ± SD and analyzed by multiple T-Test. *p < 0.05,**p < 0.01, ***p < 0.001 indicate significant difference compared to the –ve control; #*p* < 0.05, ##*p* < 0.01, ###*p* < 0.001 indicate significant difference compared to the (+ ve control); NS, no significant difference. –ve control: normal diet fed rats; HF: high fructose fed rats; H-FAT: high fat fed rats; HF/HFAT: high fat and fructose fed rats; +ve control, normal diet fed rats treated with RSV; HF-RSV: high fructose fed rats treated with RSV; HFAT-RSV: high fat fed rats treated with RSV; and HFHF-RSV: high fat and fructose fed rats treated with RSV.

The level of serum malondialdhyde (MDA) was significantly elevated and the activity of SOD in the epididymal tissue was significantly decreased in HF, HF/HFAT versus control (*p* < 0.001). The supplementation of in a dose 30 mg/kg for 4 weeks restored these abnormalities by reduction of MDA and elevation of SOD (Fig. 7).

### Effect of RSV supplementation on gene expression levels

**Fig. 8:**
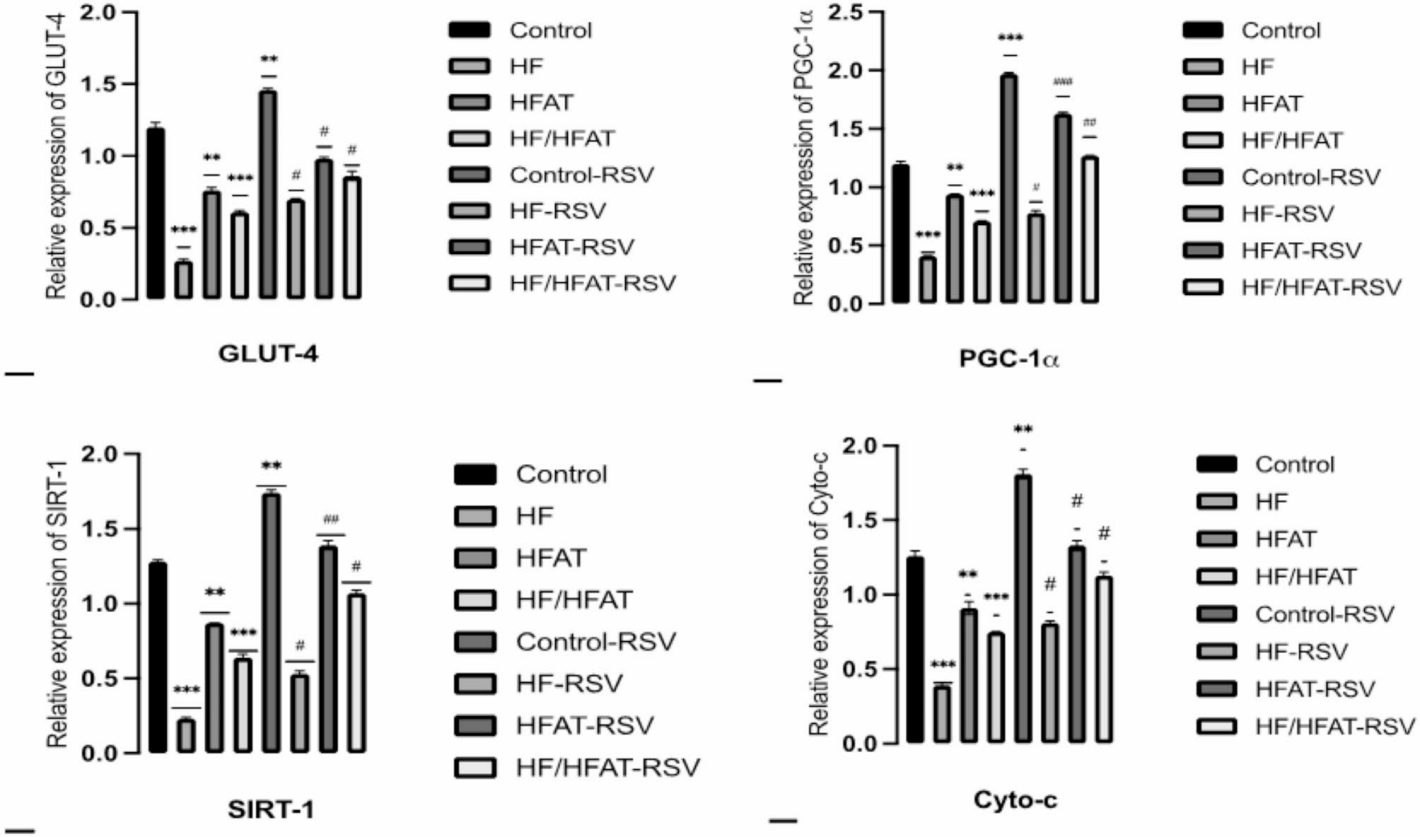
The effect of RSV on relative expression level of PGC-1α, SIRT-1, Cyto-c, and GLUT-4 in all experimental groups. Data are expressed as mean ± SD and analyzed by multiple T-Test. *p < 0.05,**p < 0.01, ***p < 0.001 indicate significant difference compared to the –ve control; #*p* < 0.05, ##*p* < 0.01, ###*p* < 0.001 indicate significant difference compared to the (+ ve control); NS, no significant difference. –ve control: normal diet fed rats; HF: high fructose fed rats; H-FAT: high fat fed rats; HF/HFAT: high fat and fructose fed rats; +ve control, normal diet fed rats treated with RSV; HF-RSV: high fructose fed rats treated with RSV; HFAT-RSV: high fat fed rats treated with RSV; and HFHF-RSV: high fat and fructose fed rats treated with RSV.

The gene expression levels of target genes were presented in Fig. 8. It was found that, the mRNA levels of PGC-1α, SIRT-1, Cyto-c, and GLUT-4 genes were significantly down regulated in HF, HFAT & HF/HFAT groups compared to control group. However, HFAT showed more downregulation of all genes. Supplementation of RSV exerted a significant upregulation of these genes compared to untreated rats (Fig. 8).

## Discussion

Obesity is a global health problem that has been increased over the last several decades. The estimated prevalence may reach to 40.4% in 2025^28^. The researches around the world were done for development a novel natural compounds help to avoid obesity. This study evaluated the mechanism of action of RSV against HF, HFAT or HF/HFAT diet induced dyslipidemia and obesity in rats.

Data obtained revealed that hypercaloric diets caused obesity in rats as evidenced by a gradual increase in body weight compared with normal diet. This observation can be explained by high fructose intake in HF or HF/HFAT models, which activate PPARc and SREBP1, two lipogenic molecules that support hepatic free fatty acids inflow, lipogenesis and weight gain^29^. Other study reported that, increased body weight gain in mice fed western diet was associated with the reduction in activity and locomotor activity^30–32^.

The HFD-induced weight gain and obesity in a rodent model was partially mediated by induction of pro-inflammatory mediators^33,34^, and the induction of brown-like adipocyte formation within white adipose tissue^35^. This is in accordance with data of current study, a significant weight loss in both HFAT or HF rat models with no impact on HF/HFAT feeding rats^36,37^. However, in male C57BL/6J mice fed HFD, RSV at a high dose of 400 mg/kg/day for 15 and 24 weeks, respectively, significantly reduced the final body weight^38,39^. This discrepancy may be due to the prolonged use of RSV high dosage supplement, indicating that weight loss may result from long-term treatment .

It was reported that, fructose consumption having been implicated in the etiology of hyperglycemia, hyperinsulinemia & insulin resistance in adult rats^40^ .This is in line with our study where the rats fed HF or HF/HFAT diets showed higher levels of fasting blood glucose, insulin and HOMA-IR than HFAT consuming rats. However, these observations contradicted with study reported that administration of 20% fructose in drinking water not implicated in the development of hyperglycemia, hyperinsulinemia, insulin resistance in adults, male & female rats^41^. In other study, the high-fat diet plus 20% (w/v) fructose in drinking water did not significantly alter glucose levels but caused hyperinsulinemia and insulin resistance in metabolic syndrome rats^42^.

Different factors could be attributed as the strain, age of the rats at the start of the study, and the route of fructose administration. Older rats are more susceptible to fructose than younger rats due to innate protective mechanisms present at an early age. Similarly, Wister rats differ from Sprague-Dawley rats to exhibit features of metabolic syndrome when fed a high-fructose diet^43,44^.

The oral administration of RSV ameliorated the alterations in glycemic parameters as results of high caloric diets consumption in HFAT or HF animal groups better than HF/HFAT animal group. Previous study demonstrated that, pretreatment with RSV caused blocking apoptosis, lowering the activation of caspase-3 and poly (adenosine diphosphate (ADP)-ribose) polymerase (PARP) in pancreatic β-cells, and prevented streptozotocin-induced diabetes in rats^45^. Other studies reported that, RSV decreased high blood glucose level in streptozotocin-nicotinamide (STZ-NA)-induced diabetic rats^46,47^. The α-cell/β-cell ratio in the pancreatic islets of rhesus monkeys was elevated after two years of resveratrol supplementation in their diet^48^. It was also found to decrease the autoimmune damage of pancreatic cells in type I diabetic animals^49^. Szkudelska et al.^50^ reported that RSV supplementation in 20 mg/kg bw reduced blood glucose level and improved glucose tolerance in Goto-Kakizaki diabetic rats^50^.

Previous studies, reported that RSV affect metabolic parameters and glycemic control depending on dosage and the duration of the intervention. With longer-term interventions, statistically significant and clinically meaningful outcomes were noted. After eight-weeks intervention, RSV supplements (800 or 1000 mg/day) significantly decreased fasting blood glucose^51,52^, but no changes were identified after five weeks intervention^53^. A small percentage of the polyphenol supplements used in the studies included frozen dried fruits and extracts, but the majority contained pure RSV in the form of capsules. The divergent results were related to bioavailability, variety, ripeness, storage, region, and environmental factors^54^.

In the current study, rats fed on HF, HFAT & HFHF diets resulted in a significant elevation in the levels of TG, TC and LDL-c versus control. Dyslipidemia is a key component of metabolic syndrome, hypertension and atherosclerosis^55^. The atherogenic index used to evaluate the risk of developing metabolic syndrome and coronary artery disease is the triglyceride to HDL-cholesterol ratio^56^. Consuming high-calorie diets for long time raised the TG/ HDL-c ratio, indicating a higher risk of metabolic syndrome and associated cardiovascular disease^57^. RSV was found to be effective treatment for the dyslipidemia induced by HF or HFAT or HF/HFAT diets. Study revealed that supplementation of polyphenolic compounds showed a significant increase in HDL-c level^51,58^. These effects might be contributed by the role of polyphenols through controlling the expression of the primary cholesterol transporters, which led to the development of mature HDL-c. This facilitates the elimination of excess cholesterol^23^. Furthermore, following RSV supplementation a significant decreased in LDL-c level^52^.

Malondialdhyde (MDA) is a marker of lipid peroxidation and oxidative damage. The higher MDA content denotes increased production of reactive oxygen species. In our study, it was found that MDA level was increased significantly in the sera of rats fed HF or HF/HFAT diets compared with control. However, RSV treatment considerably reduced MDA level significantly compared with untreated. One of explanation is its direct capability of scavenging capacity of free radicals. RSV can decrease ROS production, protecting against damage of lipids, proteins and nucleic acids. Moreover, RSV has indirect antioxidant effects, mainly covering changes in the activities of antioxidant enzymes^59^. It was found that, RSV decreased activity of SOD in adipose tissue. This enzyme catalyzes the dismutation of superoxide anion free radical (O_2_^−^) into molecular oxygen and hydrogen peroxide (H_2_O_2_) and decreases O_2_^−^ level which damages the cells at excessive concentration. Consequently, reduced enzyme activity accompanied by unchanged MDA blood level suggests a decrease in superoxide anion availability, which could be explained by RSV antioxidant property.

To investigate the mechanism of action of RSV, we focused on molecular gene expression levels involved in white adipose tissue (WAT). The nicotinamide adenine dinucleotide (NAD^+^)-dependent protein deacetylase known as sirtuin 1, which is encoded by the SIRT1 gene, exert anti-inflammatory effect in adipose tissue through direct deacetylation of NF-kB and chromatin remodeling at the promoter of inflammatory genes^60,61^. SIRT1 also acts as a cellular energy sensor and drives glucose and fat metabolism by regulating the expression of important genes, such as PPARG and UCP2^62–64^. In this study, the rats fed HF or HFAT or HF/HFAT diet showed a downregulation in SIRT1 gene compared with control rats. This alteration was alleviated by RSV supplementation in a dose 30 mg/kg for 4 weeks. As the SIRT1 was boosted via adenosine monophosphate-activated protein kinase (AMPK)^65^, SIRT1 activation is a mechanism by which RSV exert its effects through AMPK activation. Previous studies found that activating AMPK/SIRT1 pathway protects against HFD-induced obesity and may be considered as a protective effect for the management of obesity and associated metabolic complications^66,67^.

It was reported that, the RSV induced expression and function of PGC-1α, the master regulator of mitochondrial biogenesis and oxidative phosphorylation^68^. The PGC-1α gene was found to be downregulated when the animals were fed HF or HFAT or HF/HFAT diet in this study. Supplementation of RSV restored its expression level. Kleiner et al.^69^ found that downregulation of genes expression related to oxidative phosphorylation and β-oxidation, impaired glucose tolerance, and insulin resistance in adipose tissue of PGC-1α knockout (KO) mice fed a HFAT diet. Despite the low level of endogenous PGC-1α expression in WAT, this indicated that, downregulation of PGC-1α in WAT is linked to disruption of whole-body metabolism related to obesity. Mitochondrial oxidative phosphorylation are promoted by AMPK, an important energy balance regulator that can control PGC-1α expression and activity through the phosphorylation. AMPK directly phosphorylates PGC-1α, increasing its transcriptional activity, and directly affecting its expression^69^.

A SIRT1, a NAD^+^-dependent deacetylase, increased PGC-1α′s transcriptional level by deacetylation and AMPK induced SIRT1’s activity by increasing the cellular NAD^+^/NADH ratio, thereby augmented its activity^70^. Based on our data obtained, RSV acts as a positive regulator to AMPK/SIRT1/ PGC-1α axis in WAT. A study found that RSV treated rats increased aerobic performance due to the activation of the AMPK-SIRT1-PGC1α axis^71^. In another study conducted in obese patients, activated AMPK, increased SIRT1 and PGC-1α protein levels in muscles at a dose 150 mg/day for 30 days. Similar study emphasized the role of AMPK/SIRT1/PCG-1α pathway as a target of obesity treatment using other dietary products. Xu et al.,^72^demonstrated that, kaempferol (polyphenol) treated mice was rescued from diet-induced obesity by promoting white adipose browning which correlated with AMPK/SIRT1/PGC-1α pathway modulation,. However, An intriguing finding was reported, the insulin resistant mice (HFAT) supplemented with RSV for 12 weeks showed improved insulin action along with increased AMPK phosphorylation and PGC1a expression but not SIRT1^73^.

Regarding energy metabolism, the PGC-1α-NRF-1/2 pathway facilitates the activation of oxidative phosphorylation by promoting the expression of genes related to mitochondrial complexes I, II, III, IV, and cytochrome c^74^. An increase in PGC-1α expression stimulates the expression of NRF-1/2, which in turn triggers the expression of TFAM transcriptional factor, which binds to mtDNA, and initiates the transcription and replication of oxidative phosphorylation proteins. Animals fed on HF or HFAT or HF/HFAT in this study showed a decrease in cytochrome c gene expression because of downregulation of PGC-1α gene however, the RSV attenuates the expression level of cytochrome C in RSV treated groups.

It was reported that, skeletal muscle and adipose tissue account for 90% of insulin-stimulated glucose uptake^75^. Thus, the glucose transport mechanism in adipose tissue is crucial for elucidating the mechanisms that underlie the effect of RSV on glucose metabolism. The levels of PGC-1α, UCP1, and GLUT4 serve as significant indicators of glucose uptake activity and energy expenditure within adipose and muscular tissues^75,76^. In addition, PGC-1α can enhance the expression of GLUT4 and UCP1 to increase glucose uptake and metabolism^68,77^. This study demonstrated that RSV treatment significantly up-regulated PGC-1α and GLUT4 genes in the adipose tissues of rats. It was suggested that, RSV may enhance glucose uptake and utilization by activating the PGC-1α/GLUT4 pathway, thereby leading to a reduction in blood glucose levels in obese rats fed on HF or HFAT or HF/HFAT. Taken together, our data suggests that pharmacological activation of the PGC1a/GLUT4 axis with RSV enhances glucose metabolism. In line with our findings, evidence from animal studies demonstrated that RSV promotes GLUT4 expression & its translocation to the plasma membrane in insulin-resistant animals^50,78–82^.

In conclusion, supplementation of RSV at lower doses ameliorated the alterations obtained from HF or HFAT or HF/HFAT diets consumption and obesity. The antioxidant, anti-diabetic and anti-hyperlipidemic effect of resveratrol may be related to the modulation of PGC-1α, SIRT-1, Cyto-c, and GLUT-4 genes expression responsible for carbohydrates/lipid metabolism, mitochondrial biogenesis & oxidative phosphorylation.

## Electronic supplementary material

Below is the link to the electronic supplementary material.

Supplementary Material 1

Supplementary Material 2

## Data Availability

All data for this study are included in this published article.

## References

[1] WHO. World Health Organization. Obesity and overweight., 2024, [Online]. Available: https://www.who.int/news-room/factsheets/%0Adetail/obesity-and-overweight.

[2] WHO. WHO European Regional Obesity Report 2022, [Online]. (2022). Available: https://www.who.int/europe/publications/i/item/9789289057738.

[3] Zhao, X. et al. Obesity increases the severity and mortality of influenza and COVID-19: A systematic review and Meta-Analysis. *Front. Endocrinol. (Lausanne)*. 10.3389/fendo.2020.595109 (2020). 33408692 10.3389/fendo.2020.595109 PMC7779975

[4] Neto, A., Fernandes, A. & Barateiro, A. The complex relationship between obesity and neurodegenerative diseases: an updated review, *Front. Cell. Neurosci.* **17**. 10.3389/fncel.2023.1294420 (2023). 10.3389/fncel.2023.1294420 PMC10665538 38026693

[5] Juonala, M. et al. Early clinical markers of overweight/obesity onset and resolution by adolescence. *Int. J. Obes.*10.1038/s41366-019-0457-2 (2020). 10.1038/s41366-019-0457-2 31591484

[6] N. K., U. S. P. B. G. S. S. et al. C, et al. Reversal of high fat diet-induced obesity through modulating lipid metabolic enzymes and inflammatory markers expressions in rats. *Arch. Physiol. Biochem.*, (2018). 10.1080/13813455.2018.1452036 29553847

[7] Perdicaro, D. J. et al. Grape pomace reduced reperfusion arrhythmias in rats with a high-fat-fructose diet. *Food Funct.* **8** (10), 3501–3509. 10.1039/c7fo01062a (2017). 10.1039/c7fo01062a 28967023

[8] Small, L., Brandon, A. E., Turner, N. & Cooney, G. J. Modeling insulin resistance in rodents by alterations in diet: what have high-fat and high-calorie diets revealed? *Am. J. Physiol. - Endocrinol. Metab.* **314** (3), E251–E265. 10.1152/ajpendo.00337.2017 (2018). 29118016 10.1152/ajpendo.00337.2017

[9] Simoes, I. C. M. et al. Fat and sugar—a dangerous duet. A comparative review on metabolic remodeling in rodent models of nonalcoholic fatty liver disease. *Nutrients* **11** (12), 1–35. 10.3390/nu11122871 (2019). 10.3390/nu11122871 PMC6950566 31771244

[10] Xu, S., Hou, D., Liu, J. & Ji, L. Age-associated changes in Gsh s-transferase gene/proteins in livers of rats. *Redox Rep.* **23** (1), 213–218. 10.1080/13510002.2018.1546985 (2018). 30444463 10.1080/13510002.2018.1546985 PMC6748684

[11] Martínez, R. et al. A combined healthy strategy for successful weight loss, weight maintenance and improvement of hepatic lipid metabolism. *J. Nutr. Biochem.* **85**, 108456. 10.1016/j.jnutbio.2020.108456 (2020). 32810797 10.1016/j.jnutbio.2020.108456

[12] Hatware, K. V. et al. Evidence for gastroprotective, anti-inflammatory and antioxidant potential of methanolic extract of Cordia dichotoma leaves on indomethacin and stress induced gastric lesions in Wistar rats. *Biomed. Pharmacother*. 10.1016/j.biopha.2018.04.007 (2018). 29660650 10.1016/j.biopha.2018.04.007

[13] Das, R. et al. Medicinal plants used against hepatic disorders in Bangladesh: A comprehensive review. *J. Ethnopharmacol.*10.1016/j.jep.2021.114588 (2022). 34480997 10.1016/j.jep.2021.114588

[14] Sun, N. N., Wu, T. Y. & Chau, C. F. Natural dietary and herbal products in anti-obesity treatment. *Molecules*10.3390/molecules21101351 (2016). 27727194 10.3390/molecules21101351 PMC6273667

[15] Karri, S., Sharma, S., Hatware, K. & Patil, K. Natural anti-obesity agents and their therapeutic role in management of obesity: A future trend perspective. *Biomed. Pharmacotherapy*. 10.1016/j.biopha.2018.11.076 (2019). 10.1016/j.biopha.2018.11.076 30481727

[16] Colica, C. et al. A Systematic Review on Natural Antioxidant Properties of Resveratrol (2018).

[17] Planas, J. M. & C. H. & Resveratrol: A polyphenol with multiple effects. *Recent. Adv. Pharm. Sci.* **114**, 101–120 (2011).

[18] Prakash, D. & Gupta, C. Role of phytoestrogens as nutraceuticals in human health. *Pharmacologyonline* **1**, 510–523 (2011).

[19] Novelle, M. G., Wahl, D., Diéguez, C., Bernier, M. & De Cabo, R. Resveratrol supplementation: Where are we now and where should we go? *Ageing Res. Rev.* **21**, 1–15. 10.1016/j.arr.2015.01.002 (2015). 10.1016/j.arr.2015.01.002 PMC5835393 25625901

[20] Hoca, M., Becer, E., Kabadayı, H., Yücecan, S. & Vatansever, H. S. The Effect of Resveratrol and Quercetin on Epithelial-Mesenchymal Transition in Pancreatic Cancer Stem Cell. *Nutr. Cancer* **72**(7), 1231–1242. 10.1080/01635581.2019.1670853 (2020). 10.1080/01635581.2019.1670853 31595775

[21] Huang, D. D., Shi, G., Jiang, Y., Yao, C. & Zhu, C. A review on the potential of Resveratrol in prevention and therapy of diabetes and diabetic complications, *Biomed. Pharmacother.* **125**. 10.1016/j.biopha.2019.109767 (2020). 10.1016/j.biopha.2019.109767 32058210

[22] Koushki, M., Amiri-Dashatan, N., Ahmadi, N., Abbaszadeh, H. A. & Rezaei-Tavirani, M. Resveratrol: A miraculous natural compound for diseases treatment. *Food Sci. Nutr.* **6**(8), 2473–2490. 10.1002/fsn3.855 (2018). 10.1002/fsn3.855 PMC6261232 30510749

[23] Zhou, L., Xiao, X., Zhang, Q., Zheng, J. & Deng, M. Deciphering the Anti-obesity Benefits of Resveratrol: The ‘gut microbiota-adipose tissue’ axis, *Front. Endocrinol.* **10**. 10.3389/fendo.2019.00413 (2019). 10.3389/fendo.2019.00413 PMC6610334 31316465

[24] Zhu, W. et al. Effects and mechanisms of Resveratrol on the amelioration of oxidative stress and hepatic steatosis in KKAy mice. *Nutr. Metab.* **11** (1). 10.1186/1743-7075-11-35 (2014). 10.1186/1743-7075-11-35 PMC4137107 25140191

[25] Macarulla, M. T. et al. Effects of different doses of Resveratrol on body fat and serum parameters in rats fed a hypercaloric diet. *J. Physiol. Biochem.*10.1007/BF03185932 (2009). 20358350 10.1007/BF03185932

[26] Kuo, S. C., Chung, H. H., Huang, C. H. & Cheng, J. T. Decrease of hyperglycemia by syringaldehyde in diabetic rats. *Horm. Metab. Res.*10.1055/s-0033-1351274 (2014). 23918689 10.1055/s-0033-1351274

[27] Schmittgen, T. D. & Livak, K. J. Analysis of relative gene expression data using real-time quantitative PCR and the 2(-Delta Delta C(T)) method. *Methods* (2001). 10.1006/meth.2001.1262 11846609

[28] Esmat, G. et al. Obesity prevalence in adults and patients with hepatitis C: results from screening a population of 50 million in Egypt. *Egypt. Liver J.* **14** (1). 10.1186/s43066-024-00326-7 (2024).

[29] Refaat, B. et al. Vitamin D3 enhances the effects of omega-3 oils against metabolic dysfunction-associated fatty liver disease in rat. *BioFactors*. 10.1002/biof.1804 (2022). 34767670 10.1002/biof.1804

[30] Bjursell, M. et al. Acutely reduced locomotor activity is a major contributor to Western diet-induced obesity in mice. *Am. J. Physiol. - Endocrinol. Metab.* **294** (2). 10.1152/ajpendo.00401.2007 (2008). 10.1152/ajpendo.00401.2007 18029443

[31] Park, E. J. et al. Beneficial effects of lactobacillus plantarum strains on non-alcoholic fatty liver disease in high fat/high Fructose diet-fed rats. *Nutrients* **12** (2). 10.3390/nu12020542 (2020). 10.3390/nu12020542 PMC7071439 32093158

[32] Qin, L., Zhao, Y., Zhang, B. & Li, Y. Amentoflavone improves cardiovascular dysfunction and metabolic abnormalities in high Fructose and fat diet-fed rats, in Food and Function. *R. Soc. Chem.* 243–252. 10.1039/c7fo01095h (2018). 10.1039/c7fo01095h 29168869

[33] Kang, L., Heng, W., Yuan, A., Baolin, L. & Fang, H. Resveratrol modulates adipokine expression and improves insulin sensitivity in adipocytes: Relative to inhibition of inflammatory responses. *Biochimie* **92**(7), 789–796. 10.1016/j.biochi.2010.02.024 (2010). 10.1016/j.biochi.2010.02.024 20188786

[34] Zhao, Y. et al. The Beneficial Effects of Quercetin, Curcumin, and Resveratrol in Obesity. *Oxid. Med. Cell. Longev*. 10.1155/2017/1459497 (2017). 10.1155/2017/1459497 PMC5613708 29138673

[35] Wang, S. et al. Resveratrol induces brown-like adipocyte formation in white fat through activation of AMP-activated protein kinase (AMPK) α1. *Int. J. Obes.* **39**(6), 967–976. 10.1038/ijo.2015.23 (2015). 10.1038/ijo.2015.23 PMC4575949 25761413

[36] Jiang, M. et al. Oral Administration of Resveratrol Alleviates Osteoarthritis Pathology in C57BL/6J Mice Model Induced by a High-Fat Diet. *Mediat. Inflamm.*. 10.1155/2017/7659023 (2017). 10.1155/2017/7659023 PMC5303602 28250578

[37] Tian, Y. et al. Resveratrol supplement inhibited the NF-κB inflammation pathway through activating AMPKα-SIRT1 pathway in mice with fatty liver. *Mol. Cell. Biochem.*10.1007/s11010-016-2807-x (2016). 27613163 10.1007/s11010-016-2807-x

[38] Lagouge, M. et al. Resveratrol improves mitochondrial function and protects against metabolic disease by activating SIRT1 and PGC-1α. *Cell*. 10.1016/j.cell.2006.11.013 (2006). 17112576 10.1016/j.cell.2006.11.013

[39] Zhang, J. et al. The protective effect of Resveratrol on islet insulin secretion and morphology in mice on a high-fat diet. *Diabetes Res. Clin. Pract.* **97** (3), 474–482. 10.1016/j.diabres.2012.02.029 (2012). 22497970 10.1016/j.diabres.2012.02.029

[40] Ibitoye, O. B. & Ajiboye, T. O. Dietary phenolic acids reverse insulin resistance, hyperglycaemia, dyslipidaemia, inflammation and oxidative stress in high-fructose diet-induced metabolic syndrome rats. *Arch. Physiol. Biochem.* **124**(5), 410–417. 10.1080/13813455.2017.1415938 (2018). 10.1080/13813455.2017.1415938 29260581

[41] Muhammad, N., Lembede, B. W. & Erlwanger, K. H. Neonatal zingerone protects against the development of high-fructose diet-induced metabolic syndrome in adult Sprague-Dawley rats. *J. Dev. Orig. Health Dis.* **12**(4), 671–679. 10.1017/S2040174420000525 (2021). 10.1017/S2040174420000525 32500848

[42] Irfan, H. M., Khan, N. A. K. & Asmawi, M. Z. Moringa oleifera lam. Leaf extracts reverse metabolic syndrome in Sprague Dawley rats fed high-fructose high fat diet for 60-days. *Arch. Physiol. Biochem.* **128** (5), 1202–1208. 10.1080/13813455.2020.1762661 (2022). 32412306 10.1080/13813455.2020.1762661

[43] de Moura, R. F., Ribeiro, C., de Oliveira, J. A., Stevanato, E. & de Mello, M. A. R. Metabolic syndrome signs in Wistar rats submitted to differenthigh-fructose ingestion protocols. *Br. J. Nutr.* **101**, 1178–1184 (2009). 19007450 10.1017/S0007114508066774

[44] Mamikutty, N. et al. The establishment of metabolic syndrome model by induction of Fructose drinking water in male Wistar rats. *Biomed. Res. Int.*10.1155/2014/263897 (2014). 25045660 10.1155/2014/263897 PMC4086416

[45] Ku, C. R. et al. Resveratrol prevents streptozotocin induced diabetes by inhibiting the apoptosis of pancreatic beta-cell and the cleavage of Poly (ADP-ribose) Polymerase(ADP-ribose) Polymerase. *Endocr. J.* **59**, 103–109 (2012). 22068111 10.1507/endocrj.ej11-0194

[46] Palsamy, P. & Subramanian, S. Ameliorative potential of resveratrol on proinflammatory cytokines, hyperglycemia mediated oxidative stress, and pancreatic β-cell dysfunction in streptozotocin-nicotinamide-induced diabetic rats, *J. Cell. Physiol.* **224**(2), 423–432. 10.1002/jcp.22138 (2010). 10.1002/jcp.22138 20333650

[47] Soufi, F. G., Vardyani, M., Sheervalilou, R., Mohammadi, M. & Somi, M. H. Long-term treatment with Resveratrol attenuates oxidative stress pro-inflammatory mediators and apoptosis in streptozotocin-nicotinamide-induced diabetic rats. *Gen. Physiol. Biophys.* **31**(4), 431–438. 10.4149/gpb_2012_039 (2012). 23255670 10.4149/gpb_2012_039

[48] Fiori, J. L. et al. Resveratrol prevents β-cell dedifferentiation in nonhuman primates given a high-fat/high-sugar diet, *Diabetes* **62**(10), 3500–3513. 10.2337/db13-0266 (2013). 10.2337/db13-0266 PMC3781448 23884882

[49] Lee, S. M. et al. Prevention and treatment of diabetes with Resveratrol in a non-obese mouse model of type 1 diabetes. *Diabetologia* **54** (5), 1136–1146. 10.1007/s00125-011-2064-1 (2011). 10.1007/s00125-011-2064-1 PMC4036531 21340626

[50] Szkudelska, K. et al. Resveratrol affects insulin signaling in type 2 diabetic goto-kakizaki rats. *Int. J. Mol. Sci.* **22**(5), 1–15. 10.3390/ijms22052469 (2021). 10.3390/ijms22052469 PMC7957525 33671110

[51] Abdollahi, S. et al. Dec., The Effect of Resveratrol Supplementation on Cardio-Metabolic Risk Factors in Patients with Type 2 Diabetes: A Randomized, Double-Blind Controlled Trial. *Phyther. Res.* **33**(12), 3153–3162. 10.1002/ptr.6487 (2019). 10.1002/ptr.6487 31475415

[52] Khodabandehloo, H., Seyyedebrahimi, S. S., Esfahani, E. N., Razi, F. & Meshkani, R. Resveratrol supplementation decreases blood glucose without changing the circulating CD14 + CD16 + monocytes and inflammatory cytokines in patients with type 2 diabetes: a randomized, double-blind, placebo-controlled study. *Nutr. Res.* **54**, 40–51. 10.1016/j.nutres.2018.03.015 (2018). 10.1016/j.nutres.2018.03.015 29914666

[53] Thazhath, S. S. et al. Administration of Resveratrol for 5 Wk has no effect on glucagon-like peptide 1 secretion, gastric emptying, or glycemic control in type 2 diabetes: A randomized controlled trial. *Am. J. Clin. Nutr.* **103** (1), 66–70. 10.3945/ajcn.115.117440 (2016). 10.3945/ajcn.115.117440 26607942

[54] Castro-Barquero, S. et al. Mar., Dietary polyphenol intake is associated with HDL-cholesterol and a better profile of other components of the metabolic syndrome: A PREDIMED-plus sub-study, *Nutrients* **12**(3). 10.3390/nu12030689 (2020). 10.3390/nu12030689 PMC7146338 32143308

[55] Hannou, S. A., Haslam, D. E., McKeown, N. M. & Herman, M. A. Fructose metabolism and metabolic disease. *J. Clin. Invest.* **128** (01), 545–555. 10.1172/JCI96702 (2018). no. 2American Society for Clinical Investigation. 29388924 10.1172/JCI96702 PMC5785258

[56] Da Luz, P. L., Favarato, D., Faria-Neto, J. R., Lemos, P. & Chagas, A. C. P. High ratio of triglycerides to HDL-cholesterol predicts extensive coronary disease. *Clinics* **63** (4), 427–432. 10.1590/S1807-59322008000400003 (2008). 18719750 10.1590/S1807-59322008000400003 PMC2664115

[57] Chu, S. Y., Jung, J. H., Park, M. J. & Kim, S. H. Risk assessment of metabolic syndrome in adolescents using the triglyceride/high-density lipoprotein cholesterol ratio and the total cholesterol/high-density lipoprotein cholesterol ratio, *Ann. Pediatr. Endocrinol. Metab.* **24**(1), 41–48. 10.6065/apem.2019.24.1.41 (2019). 10.6065/apem.2019.24.1.41 PMC6449623 30943679

[58] Vincenzo et al. Hypoglycemic and hypolipemic effects of a new lecithin formulation of Bergamot polyphenolic fraction: A double blind, randomized, placebo-controlled study. *Endocr. Metab. Immune Disord Drug Targets*. **19**, 136–143 (2019). 30501605 10.2174/1871530319666181203151513

[59] Truong, V. L., Jun, M. & Jeong, W. S. Role of resveratrol in regulation of cellular defense systems against oxidative stress, *BioFactors*. 10.1002/biof.1399 (2018). 10.1002/biof.1399 29193412

[60] Kotas, M. E., Gorecki, M. C. & Gillum, M. P. Sirtuin-1 is a nutrientdependent modulator of inflammation. *Adipocyte* **2** (2), 113–118 (2013). 23805409 10.4161/adip.23437 PMC3661114

[61] Moreno-Navarrete, J. M. et al. DBC1 is involved in adipocyte inflammation and is a possible marker of human adipose tissue senescence. *Obesity*10.1002/oby.20999 (2015). 25682741 10.1002/oby.20999

[62] Picard, F. et al. Sirt1 promotes fat mobilization in white adipocytes by repressing PPAR-γ. *Nature*10.1038/nature02583 (2004). 15175761 10.1038/nature02583 PMC2820247

[63] Bordone, L. et al. Sirt1 regulates insulin secretion by repressing UCP2 in pancreatic Β cells. *PLoS Biol.*10.1371/journal.pbio.0040031 (2006). 16366736 10.1371/journal.pbio.0040031 PMC1318478

[64] Chaudhary, N. & Pfluger, P. T. Metabolic benefits from Sirt1 and Sirt1 activators. *Curr. Opin. Clin. Nutr. Metab. Care*. 10.1097/MCO.0b013e32832cdaae (2009). 19474719 10.1097/MCO.0b013e32832cdaae

[65] Yang, Y. et al. Regulation of SIRT1 and its roles in inflammation. *Front. Immunol.*10.3389/fimmu.2022.831168 (2022). 35359990 10.3389/fimmu.2022.831168 PMC8962665

[66] Chang, E. & Kim, Y. Vitamin D insufficiency exacerbates adipose tissue macrophage infiltration and decreases AMPK/SIRT1 activity in obese rats. *Nutrients*10.3390/nu9040338 (2017). 28353634 10.3390/nu9040338 PMC5409677

[67] Lai, J. et al. Activation of AMP-Activated protein Kinase-Sirtuin 1 pathway contributes to Salvianolic acid A-Induced Browning of white adipose tissue in High-Fat diet fed male mice. *Front. Pharmacol.*10.3389/fphar.2021.614406 (2021). 34122060 10.3389/fphar.2021.614406 PMC8193940

[68] Puigserver, P. et al. A cold-inducible coactivator of nuclear receptors linked to adaptive thermogenesis. *Cell*10.1016/S0092-8674(00)81410-5 (1998). 9529258 10.1016/s0092-8674(00)81410-5

[69] Herzig, S. & Shaw, R. J. Guardian of metabolism and mitochondrial homeostasis. *Nat. Rev. Mol. Cell Biol.*10.1038/nrm.2017.95 (2018). 28974774 10.1038/nrm.2017.95 PMC5780224

[70] Xu, Y. et al. Berberine modulates deacetylation of Pparγ to promote adipose tissue remodeling and thermogenesis via ampk/sirt1 pathway. *Int. J. Biol. Sci.*10.7150/ijbs.62556 (2021). 34421358 10.7150/ijbs.62556 PMC8375237

[71] Hart, N. et al. Resveratrol enhances exercise training responses in rats selectively bred for high running performance. *Food Chem. Toxicol.*10.1016/j.fct.2013.01.051 (2013). 23422033 10.1016/j.fct.2013.01.051 PMC3703486

[72] Xu, C. et al. Dietary kaempferol exerts anti-obesity effects by inducing the browing of white adipocytes via the AMPK/SIRT1/PGC-1α signaling pathway, *Curr. Res. Food Sci.* **8**, 100728. 10.1016/j.crfs.2024.100728 (2024). 10.1016/j.crfs.2024.100728 PMC10990952 38577419

[73] Um, J. H. et al. AMP-activated protein kinase-deficient mice are resistant to the metabolic effects of Resveratrol. *Diabetes* **59** (3), 554–563. 10.2337/db09-0482 (2010). 19934007 10.2337/db09-0482 PMC2828647

[74] Taherzadeh-Fard, E. et al. PGC-1alpha downstream transcription factors NRF-1 and TFAM are genetic modifiers of huntington disease. *Mol. Neurodegener*. 10.1186/1750-1326-6-32 (2011). 21595933 10.1186/1750-1326-6-32 PMC3117738

[75] Leto, D. & Saltiel, A. R. Regulation of glucose transport by insulin: traffic control of GLUT4. *Nat. Rev. Mol. Cell Biol.*10.1038/nrm3351 (2012). 22617471 10.1038/nrm3351

[76] Wu, Z. et al. Mechanisms controlling mitochondrial biogenesis and respiration through the thermogenic coactivator PGC-1. *Cell*10.1016/S0092-8674(00)80611-X (1999). 10412986 10.1016/S0092-8674(00)80611-X

[77] Benton, C. R. et al. Increased levels of peroxisome proliferator-activated receptor gamma, coactivator 1 alpha (PGC-1α) improve lipid utilisation, insulin signalling and glucose transport in skeletal muscle of lean and insulin-resistant obese Zucker rats. *Diabetologia* **53** (9), 2008–2019. 10.1007/s00125-010-1773-1 (2010). 20490453 10.1007/s00125-010-1773-1

[78] Deng, J. Y., Hsieh, P. S., Huang, J. P., Lu, L. S. & Hung, L. M. Activation of Estrogen receptor is crucial for resveratrol-stimulating muscular glucose uptake via both insulin-dependent and -independent pathways. *Diabetes*10.2337/db07-1750 (2008). 18426865 10.2337/db07-1750 PMC2453636

[79] Chen, L. L. et al. Resveratrol attenuates high-fat diet-induced insulin resistance by influencing skeletal muscle lipid transport and subsarcolemmal mitochondrial β-oxidation. *Metabolism*10.1016/j.metabol.2011.04.002 (2011). 21632075 10.1016/j.metabol.2011.04.002

[80] Tan, Z. et al. Caveolin-3 is involved in the protection of Resveratrol against high-fat-diet-induced insulin resistance by promoting GLUT4 translocation to the plasma membrane in skeletal muscle of ovariectomized rats. *J. Nutr. Biochem.*10.1016/j.jnutbio.2011.12.003 (2012). 22569348 10.1016/j.jnutbio.2011.12.003

[81] Yonamine, C. Y. et al. Resveratrol improves glycemic control in type 2 diabetic obese mice by regulating glucose transporter expression in skeletal muscle and liver. *Molecules*10.3390/molecules22071180 (2017). 28708105 10.3390/molecules22071180 PMC6152102

[82] Vlavcheski, F., Den Hartogh, D. J., Giacca, A. & Tsiani, E. Amelioration of high-insulin-induced skeletal muscle cell insulin resistance by Resveratrol is linked to activation of AMPK and restoration of GLUT4 translocation. *Nutrients*10.3390/nu12040914 (2020). 32230718 10.3390/nu12040914 PMC7230755

