# Supplementary material for: Role of trans-resveratrol in ameliorating biochemical and molecular alterations in obese rats induced by a high fructose/fat diet
Source: Sci Rep. 2025 Mar 6;15:7879. doi: 10.1038/s41598-025-91027-7 (PMC11885455; doi:10.1038/s41598-025-91027-7)
Supplement: Supplementary file 1 — Supplementary Material 1 [file 41598_2025_91027_MOESM1_ESM.pdf]

Response to reviewers' comments

Dear editor.

Regarding to manuscript ID: f401ed8a-bb4b-4cc1-9e41-cb59470fc72c

Title "Role of *trans*-resveratrol in ameliorating the biochemical and molecular alterations in obese rats induced by a high fructose/fat diet".

We would like to appreciate your great efforts and the reviewers for giving valuable comments, which resulted in improvement of the paper. We have done the corrections as requested by each reviewer in the research article.

Regards

Prof. Said Moselhy.

<u>Response to Reviewer I.</u>

The authors would like to thanks reviewer's for his effort and valuable comments:

Manuscript title: Role of trans-resveratrol in ameliorating the biochemical and molecular alterations in obese rats induced by a high fructose/fat diet

Major:

1. No mechanistic insights is the major weakness of this paper.

Response: This is the main aim of current study, we investigated the Mechanism of action of resveratrol as explained in discussion section (pages 7 and 8) Via SIRT 1/PCG-α axis & PCG-α/GLUT-4 axis.

2. Several typo errors and mismatches of Figures should be carefully revised throughout the manuscript.

Response: All figures has been carefully revised.

3. Page 3 line 11" Forty male Wistar rats (weighing 180-200g) were obtained from VACSERA (Cairo, Egypt)". But the weight of rats in the 1st month of HF, HFAT, HF/HFAT was only 113.9g (control). Why is the weight of 1st month lower than the starting weight?

Response: Sorry for this mistyping, in page 2, the Starting weight of rats was (113.9-136.3).

4. The treatment duration of HF, HFAT, HF/HFAT and subsequent RSV should indicated clearly in method section (HF, HFAT, HF/HFAT for 6 months and follow RSV 1 month).

Response: It was clarified in details in the materials & methods section (page 3). All rats received the diets daily according to their group for 24 weeks then subsequent RSV supplementation for 4 weeks.

5. Page 3 line 31 "After 6 months of the experiment, the animals fasted overnight before blood and tissue collection & were sacrificed by decapitation under anesthesia with thiopental (15 mg/kg)". No RSV treatment?

Response: Page 3: At the end of the experiment, the animals were fasted overnight. Blood and tissue were collected after scarification by decapitation under anesthesia with thiopental (15 mg/kg). The blood and tissues were collected from groups including treated with RSV.

6. NoFigure4isfound.

Response: Figure 4 was added to the figures section.

Minor:

1. The number of animal should be carefully mentioned in the method section or in the Figure legends.

Response: Page 3: The total numbers of animals & number of animals per each group are mentioned in the methods section (2.1 Experimental animal groupings and treatment). Rats were randomly divided into eight groups with 12 animals for each: group 1 (Control): Rats were received normal diet, group 2 (HF): Rats were received standard diet and 25% fructose in drinking water, group 3 (HFAT): Rats were received high fat diet, group 4 (HF/HFAT): Rats were received high fat diet & 25% fructose in drinking water, group 5 (Control-RSV): Rats were received normal diet and given orally 30 mg/Kg BW of RSV, group 6 (HF-RSV): Rats were received normal diet and 25% fructose in drinking water with oral doses of (30 mg/Kg b.w) of RSV, group 7 (HFAT-RSV): Rats were received high fat diet with oral doses of 30 mg/Kg bw of RSV & group 8 (HF/HFAT-RSV): Rats were received high fat diet & 25% fructose in drinking water with oral doses of 30 mg/Kg b.w of RSV. The dose of RSV was given according to Macarulla etal.,(2009).

2. Page 5 line 2. ….. as cataract and skin ulcer (figure 1.3), it should be (Figure3)

Response: It was corrected.

3. Figure 5, it is FBG not FBS.

Response: It was changed.

Response to Reviewer II:

The authors would like the thanks the reviewer for great effort and comments.

1) Language and Presentation: The manuscript requires significant improvement in language and style to ensure clarity and readability

Response: The manuscript was carefully revised for English language editing. A copy of revised manuscript with track changes corrections was attached.

2) Introduction: Why this study is conducted in animal model, while Clinical trials were conducted using varying dosages, durations, and methods of administration of resveratrol?

Response: The study was conducted to identify the mechanism of action of resveratrol through signal pathway axis: PGC-1α, SIRT-1, Cyto-c, and GLUT-4 genes. This will open a new window in management protocol of obesity.

3) What is the rational for using the High fat, High fructose and the combinations of these two diets? What is the aims of authors in designing such obesity model? Please explain in more details in introduction section.

Response: It was explained in details in the introduction section, In page 2, second paragraph staring with (Experimental animal models are indispensable tools for investigating the changes in morphology and metabolic pathways that contribute to the onset of overweight and obesity. Diet-induced obesity (DIO) animal model may be more accurate to the actual scenario that exists in the human population. Typically, a high-fat semi-synthetic diet (HFD) with 45–60% of total calories is used to create DIO models. It was reported that, the use of diets with 45 % of kcal present as fat in combination with a high mono or di-saccharide content (fructose or sucrose) has been advocated for DIO models (Simoes et al., 2019; Small et al., 2018). The experimental animal period may affect different organ functionality caused by the dietary components for development of obesity (Martínez et al., 2020 and Xu et al., 2018).

4) In Experimental animal groupings and treatment, the authors have mentioned that there are three groups in the study:" After one-week of acclimation, rats were randomly divided into three groups with 8 animals for each group treated as follows" while there were 8 groups in the study. Please, explain this paradox in material method section.

Response: Page 3:The animals grouping and number of each group was clarified in materials & methods section.(Rats were randomly divided into eight groups with 12 animals for each: group 1 (Control): Rats were received normal diet, group 2 (HF): Rats were received standard diet and 25% fructose in drinking water, group 3 (HFAT): Rats were received high fat diet, group 4 (HF/HFAT): Rats were received high fat diet & 25% fructose in drinking water, group 5 (Control-RSV): Rats were received normal diet and given orally 30 mg/Kg BW of RSV, group 6 (HF-RSV): Rats were received normal diet and 25% fructose in drinking water with oral doses of (30 mg/Kg b.w) of RSV, group 7 (HFAT-RSV): Rats were received high fat diet with oral doses of 30 mg/Kg bw of RSV & group 8 (HF/HFAT-RSV): Rats were received high fat diet & 25% fructose in drinking water with oral doses of 30 mg/Kg b.w of RSV. The dose of RSV was given according to Macarulla etal.,(2009).

5) Explain how the sample size was decided? Provide details of any a priori sample size calculation, if done.

Response: As the experiment lasting for six months, the sample size was not calculated

6) How and why the doses of 30 mg/Kg bw of resveratrol was chosen for treatment?

Response: The dose of RSV was given according to Macarulla etal.,(2009) as mentioned in experimental animals section (page 3).

7) How long is the treatment period? In abstract section the authors have mentioned 4 weeks but in method part the 6 months of experiment was conducted.

Response: Page 3:The total experimental period was 7 months that were divided into 24 weeks for obesity development and 4 weeks for RSV supplementation.

8) State whether randomization was used to allocate experimental units to control and treatment groups. If done, provide the method used to generate the randomization sequence.

Response: The randomization to avoid any bias in the experiment data from any unknown factor. No specific method used for this issue.

9) Please explain how the High fat diet was prepared? and What was the source of fat to prepare the High Fat Diet?

Response: It was mentioned in material section : The HFD was prepared according to diet formula (D12451) by mixing (per 100g diet; 24 g protein, 41 g carbohydrate, 24g fat).

10) Please give detailed information about the exact number of experimental units allocated to each group, and the total number in each experiment. Also indicate the total number of animals used. It is not clear how many groups are there and also the number of animals in each groups?

Response: The number of groups was 8 groups, each group included 12 rats. This was clarified in materials & methods section

(Rats were randomly divided into eight groups with 12 animals for each: **group 1 (Control)**: Rats were received normal diet, **group 2 (HF):** Rats were received standard diet and 25% fructose in drinking water, **group 3 (HFAT):** Rats were received high fat diet, **group 4 (HF/HFAT):** Rats were received high fat diet & 25% fructose in drinking water, **group 5 (Control-RSV):** Rats were received normal diet and given orally 30 mg/Kg BW of RSV, **group 6 (HF-RSV):** Rats were received normal diet and 25% fructose in drinking water with oral doses of (30 mg/Kg b.w) of RSV, **group 7 (HFAT-RSV)**: Rats were received high fat diet with oral doses of 30 mg/Kg b.w of RSV & **group 8 (HF/HFAT-RSV)**: Rats were received high fat diet & 25% fructose in drinking water with oral doses of 30 mg/Kg b.w of RSV. The dose of RSV was given according to Macarulla etal.,(2009).

11) Describe the strategy used to minimize potential confounders. If confounders were not controlled, state this explicit.

Response: No specific strategy, but to minimize the confounders, random selection of animals groups was done to avoid any bias in the experiment data from any unknown factor
